# Angiotensin-II Drives Human Satellite Cells Toward Hypertrophy and Myofibroblast Trans-Differentiation by Two Independent Pathways

**DOI:** 10.3390/ijms20194912

**Published:** 2019-10-03

**Authors:** Annunziatina Laurino, Valentina Spinelli, Manuela Gencarelli, Valentina Balducci, Leonardo Dini, Lorenzo Diolaiuti, Marco Ghionzoli, Antonio Messineo, Alessandro Mugelli, Elisabetta Cerbai, Laura Raimondi, Laura Sartiani

**Affiliations:** 1 Department of NEUROFARBA, Center of Molecular Medicine, University of Firenze, 50139 Firenze, Italy; annunziatina.laurino@unifi.it (A.L.); valentina.spinelli@unifi.it (V.S.); manuela.gencarelli@unifi.it (M.G.); valentina.balducci@unifi.it (V.B.); dini.leonardo88@gmail.com (L.D.); lorenzod88@live.it (L.D.); marco.ghionzoli@meyer.it (M.G.); antonio.messineo@meyer.it (A.M.); alessandro.mugelli@unifi.it (A.M.); elisabetta.cerbai@unifi.it (E.C.); laura.raimondi@unifi.it (L.R.); 2 Department of Pediatric Surgery, Meyer Children’s University Hospital, 50139 Firenze, Italy

**Keywords:** satellite cells, angiotensin, TRPC channels, Ca^2+^ signaling, myofibroblast, myogenesis, hypertrophy, skeletal muscle

## Abstract

Skeletal muscle regeneration is ensured by satellite cells (SC), which upon activation undergo self-renewal and myogenesis. The correct sequence of healing events may be offset by inflammatory and/or fibrotic factors able to promote fibrosis and consequent muscle wasting. Angiotensin-II (Ang) is an effector peptide of the renin angiotensin system (RAS), of which the direct role in human SCs (hSCs) is still controversial. Based on the hypertrophic and fibrogenic effects of Ang via transient receptor potential canonical (TRPC) channels in cardiac and renal tissues, we hypothesized a similar axis in hSCs. Toward this aim, we demonstrated that hSCs respond to acute Ang stimulation, dose-dependently enhancing p-mTOR, p-AKT, p-ERK1/2 and p-P38. Additionally, sub-acute Ang conditioning increased cell size and promoted trans-differentiation into myofibroblasts. To provide a mechanistic hypothesis on TRPC channel involvement in the processes, we proved that TRPC channels mediate a basal calcium entry into hSCs that is stimulated by acute Ang and strongly amplified by sub-chronic Ang conditioning. Altogether, these findings demonstrate that Ang induces a fate shift of hSCs into myofibroblasts and provide a basis to support a benefit of RAS and TRPC channel blockade to oppose muscle fibrosis.

## 1. Introduction

Healthy skeletal muscle has a powerful regenerative potential that enables physiological growth and efficient repair following insults. Conditions able to impair this capacity have a negative prognostic impact on disability by promoting skeletal muscle frailty [1].

Muscle regeneration is ensured by resident stem cells, i.e., satellite cells (SCs), which at rest lie quiescently in niches adherent to myofibrils. Following injuries, SCs are prompted to activate and migrate into the damaged area, eventually entering into symmetric and asymmetric divisions aimed to replenish the SC pool and accomplish regenerative myogenesis [1]. SC activation and differentiation are finely orchestrated events, largely dependent on niche composition; in this respect, enrichment of the local microenvironment with inflammatory and/or fibrotic factors may drive SCs toward incorrect healing and consequent muscle wasting [2]. The identification of factors and mechanisms inducing muscle fibrosis may help uncover innovative drug targets potentially useful to prevent or limit muscle deterioration.

Angiotensin II (Ang) is one of the effector peptides of the renin angiotensin system (RAS), of which the role in skeletal muscle, and particularly on SCs, remains controversial. Compelling studies have demonstrated detrimental effects of Ang on mouse SC growth and activation [2,3], while others are suggestive of positive effects of Ang on muscle healing via chemiotaxis of C2C12 myoblasts [4,5]. Interestingly, Yoshida et al. [6] revealed a positive role of Ang type-2 (AT2) receptors in skeletal muscle regeneration. Regardless of the prominent effects, evidence in cardiovascular patients indicates that long-term therapies with angiotensin-converting enzyme inhibitors (ACEIs) or Ang type-1 (AT1) receptor antagonists (ARBs) [7,8] slow down the progressive decline of muscle mass and contractile performance, often associated with cardiac and renal hypertrophy and fibrosis [9,10,11,12,13,14,15,16,17]. However, it is not clear whether ACEIs or ARBs ameliorate muscle wasting through the benefits exerted at cardiac and renal levels or whether they also directly enhance muscle regenerative capacity and/or its resistance to injuries. Of note, there is limited knowledge on the effects of Ang and ARBs on human SCs (hSCs), a setting in which the presence of a complete RAS has not been documented in detail [18,19].

In cardiac and renal pathologies, over-activation of RAS conveys distinct pathogenic signals responsible for inflammation, hypertrophy, and fibrosis [7,8,20]. Some of these effects are secondary to Ang receptor activation and subsequent stimulation of the canonical transient receptor potential (TRPC) channels [21,22,23,24,25,26,27], evidence that attributes a distinctive role to the Ang-TRPC channel axis in cardio-renal hypertrophy and fibrosis. TRPC channels comprise seven different isoforms (TRPC1–7) co-assembling to form non-selective, Ca^2+^-permeable tetramers [28]. They underlie the store-operated channels, being activated by depletion of intracellular Ca^2+^ stores; additionally, they can be activated by mechanical stretch and by stimulation of Gq-coupled receptors, including Ang receptors [21,22,23,24,25,26,27]. Since Ca^2+^ dynamics regulates skeletal muscle development, homeostasis, and regeneration [29,30], we hypothesized a possible role of Ang-TRPC channel axis on hSC fate determination toward non-myogenic lineage. To test this hypothesis, in this study, we provided evidence that Ang promotes hypertrophy and myofibroblast trans-differentiation of hSCs and that this fate decision is associated to Ca^2+^ entry via activation of TRPC channels. In parallel, to elucidate the mechanisms and provide a basis to support clinical benefits of RAS blockade in muscle fibrosis and wasting, the effects of Ang were evaluated in the presence of irbesartan (Irb), an AT1 receptor antagonist.

## 2. Results

### 2.1. Characterization of hSC Phenotype

**Figure 1:**
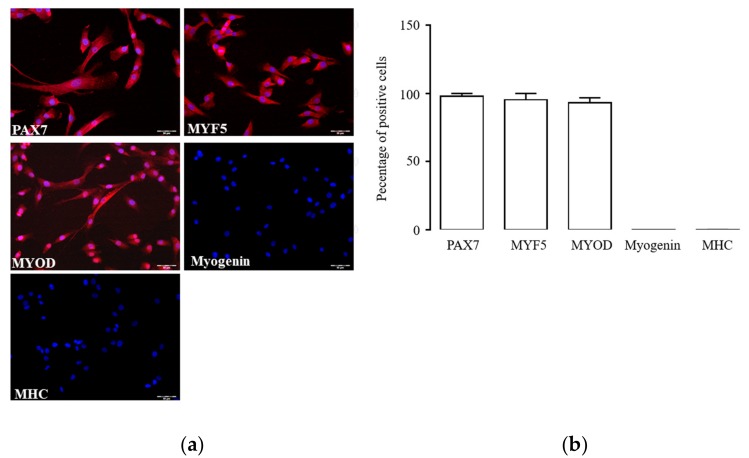
Phenotype of cultured human satellite cells (hSCs): immunolabelling (**a**) and percentage of positive cells (**b**) for Paired box protein 7 (PAX7), myogenic factor 5 (MYF5), myogenic factor 3 (MYOD), myogenin, and myosin heavy chain (MHC) in cultured hSCs at passage 3. Target proteins are depicted in red, and cell nuclei are in blue (DAPI). The image magnification is 20×.

Primary hSCs undergo quick phenotype modifications in culture that may potentially interfere with experimental findings. To overcome this limitation, we first identified the passage interval of cell phenotype stability by immunofluorescence detection of key myogenic markers. In our experimental conditions, hSCs at passages 1–3 expressed Paired box protein 7 (PAX7, Figure 1a), a transcription factor present in quiescent and activated SCs that is downregulated during myogenic differentiation [31]. Cells also stained positive for myogenic factor 5 (MYF5) and myogenic factor 3 (MYOD), two transcription factors of myogenic lineage [32]; at the same time, they were negative for the transcription factor myogenin and the contractile protein myosin heavy chain (MHC), indicating the absence of myogenic differentiation. While Pax7, Myf5, and MyoD were present in almost all cells at passages 1–3, Myogenin and myosin heavy chain (MHC) were rarely observable (Figure 1b). This expression pattern, typical of activated hSCs appeared stable throughout passages 1–3; thereafter, it spontaneously changed over subsequent passages, thus limiting culture use to passages 1–3 for the following experiments.

### 2.2. Activated hSCs Express a Functional RAS Protein Panel

**Figure 2:**
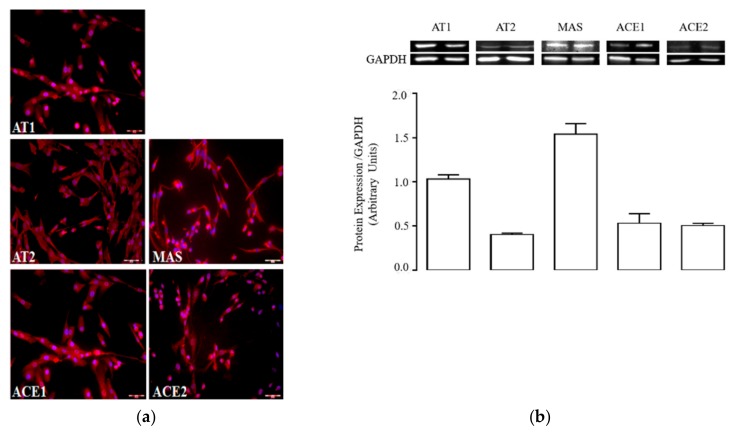
Expression of renin angiotensin system (RAS) proteins in activated hSCs: (**a**) immunolabelling of type-1 (AT1), type-2 (AT2), and MAS receptors, and angiotensin-converting enzymes 1 (ACE1) and 2 (ACE2) in cultured hSCs at passage 3. Target proteins are depicted in red, cell nuclei are in blue (DAPI). The image magnification is 20×; (**b**) immunoblotting (top) and densitometric analysis (bottom) of AT1, AT2, and MAS receptors, and ACE1 and ACE2 in protein extracts of hSCs at passage 3. The histogram shows the ratios (mean ± standard error of the mean (SEM), *n* = 3) between the expression levels of target proteins and GAPDH.

The presence of RAS protein in activated hSCs was investigated by immunofluorescence and western blot analysis of protein extracts. All individual proteins of the conventional and non-conventional RAS were expressed (Figure 2a,b); in particular, staining revealed that almost all cells were positive for AT1, AT2, and MAS receptors, which showed cytosolic and plasma membrane localization. Densitometric analysis of immunoblotting evidenced a prominent expression of MAS and AT1 over AT2 receptors in total protein extracts (Figure 2b). This expression pattern was qualitatively similar to that retrieved in whole skeletal muscle extracts (Figure S1a,b).

Type-1 and -2 angiotensin-converting enzymes (ACE1 and ACE2, respectively) were also expressed at a protein level in activated hSCs, displaying comparable amounts (Figure 2a,b). This suggests that activated hSCs retain all the enzymatic requirements necessary to synthesize Ang and possibly other related peptides.

**Figure 3:**
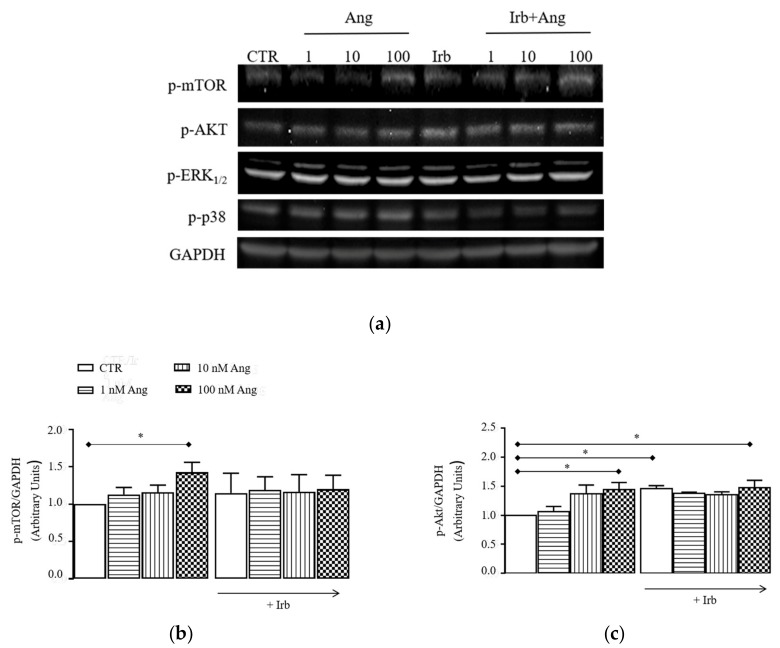

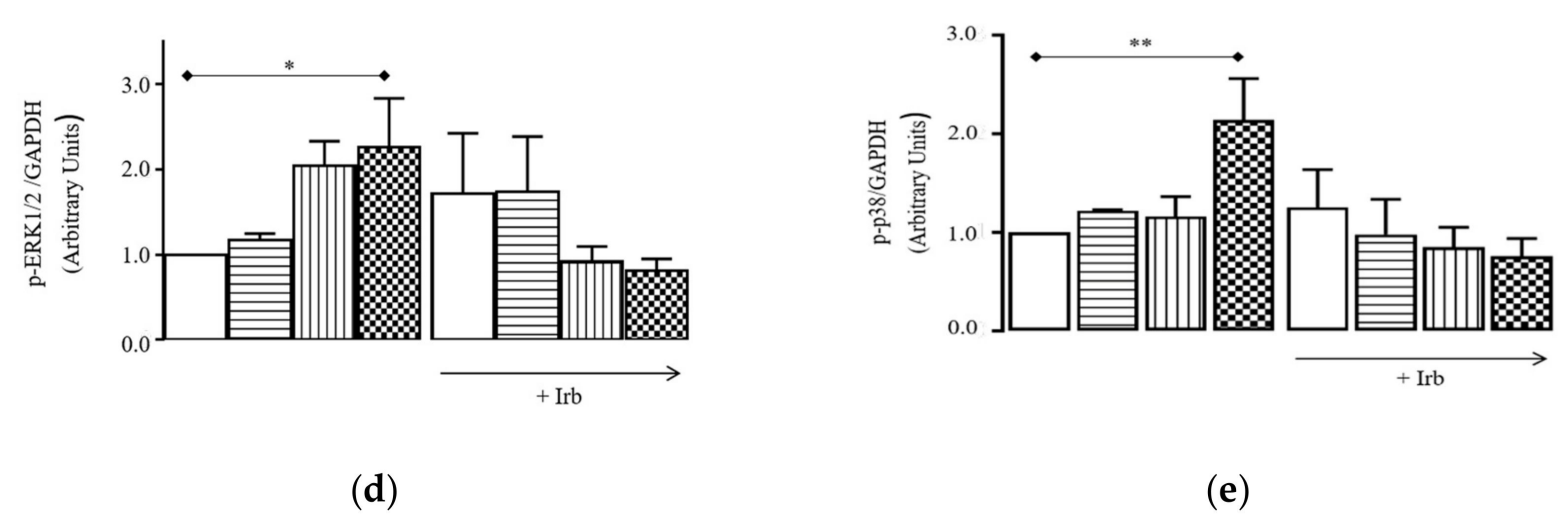
Signaling cascade stimulated by acute Ang in hSCs: (**a**) representative immunoblotting of p-mTOR, p-AKT, p-ERK1/2, p-P38, and GADPH detected in protein extracts of cultured hSCs stimulated or not (CTR) with 1–100 nM angiotensin II (Ang) for 15 min with/without 1 µM irbesartan (Irb); densitometric analysis of p-mTOR (**b**), p-AKT (**c**), p-ERK1/2 (**d**), and p-P38 (**e**) levels, reported as ratios between the levels of the target protein and GAPDH. Results are presented as mean ± SEM of the three gels. * *p* < 0.05 vs. CTR, ** *p* < 0.01 vs. CTR.

To assess the function of RAS protein in activated hSCs, we exposed cells to increasing concentrations of Ang (1, 10, and 100 nM) or standard growing medium (CTR) for 15 min and analyzed the phosphorylation levels of intracellular protein kinases that are typically enhanced by Ang in other cells, namely p-mTOR, p-AKT, p-ERK1/2, and p-P38. Results (Figure 3a–e) showed that Ang induced a concentration-dependent increase of the phosphorylation levels of each individual protein kinases, which were significant at 100 nM Ang compared to CTR (p-mTOR, p-AKT, and p-ERK1/2: * *p* < 0.05 vs. CTR; *p*-P38: ** *p* < 0.01 vs. CTR). For all proteins, enhancement of phosphorylation was similarly prevented by 1µM Irb, suggesting the involvement of AT1 receptor in these signals.

### 2.3. Sub-Chronic Conditioning of Activated hSCs with Ang Leads to Hypertrophy and Myofibroblast Trans-Differentiation Without Modification of Cell Viability

**Figure 4:**
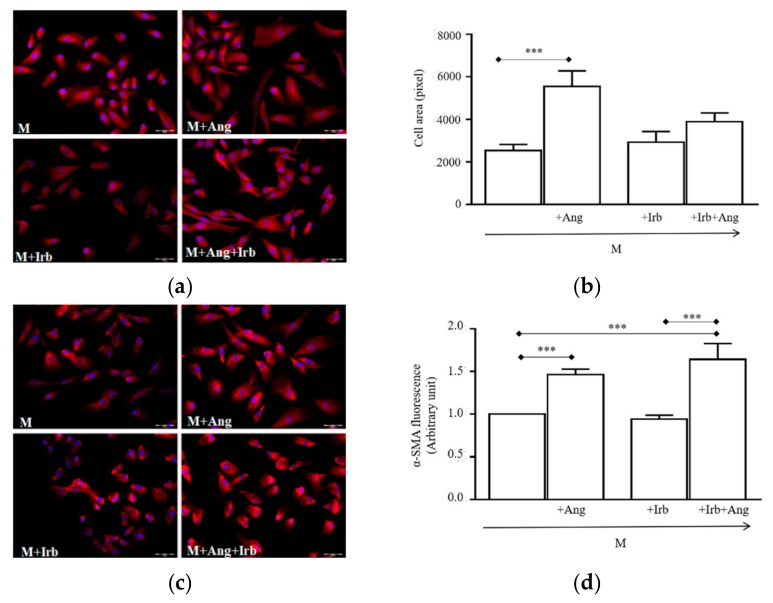

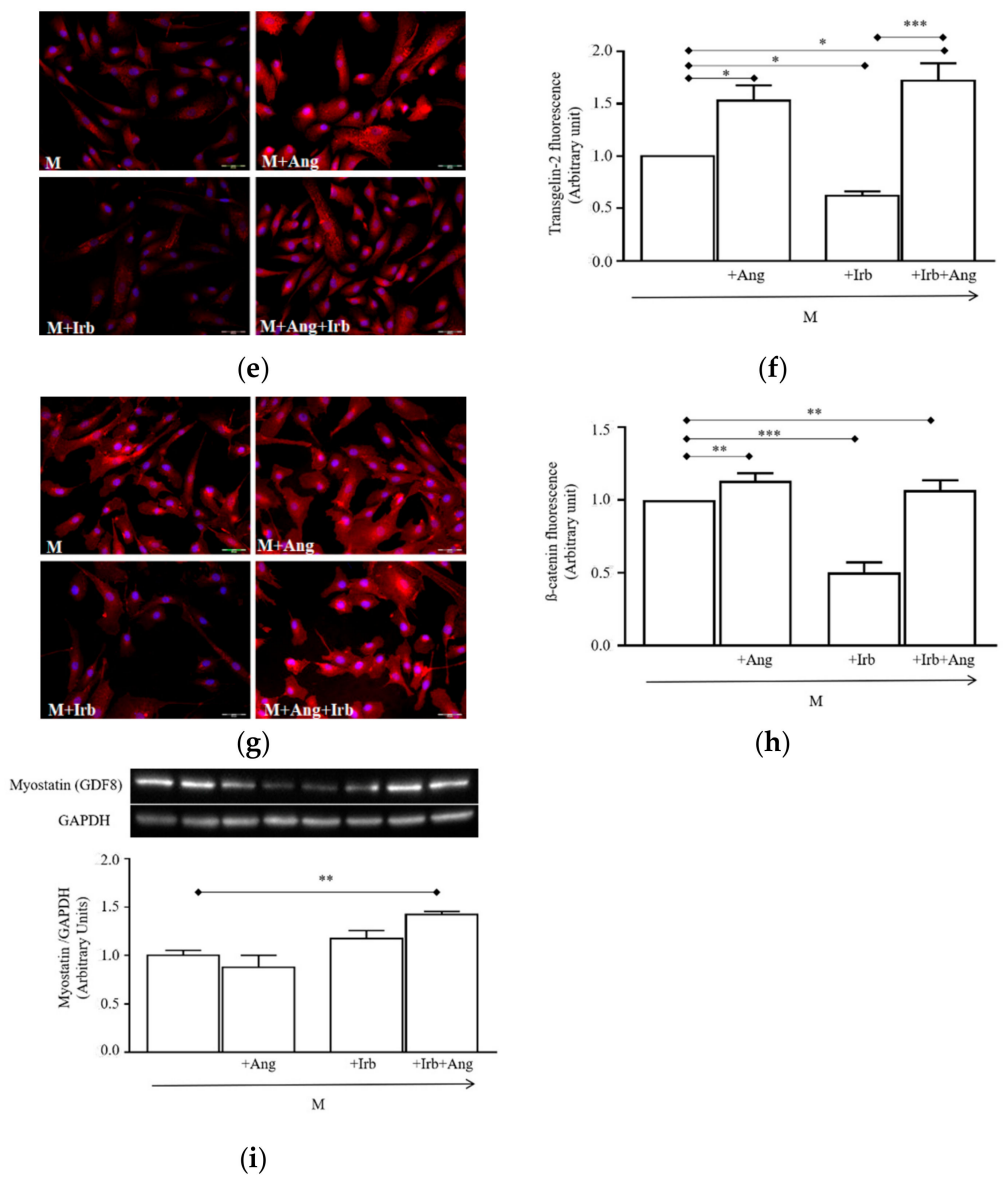
Effect of sub-chronic conditioning with Ang on hSC-size and myofibroblast markers: (**a**) Representative photomicrographs of cells labelled with Alexa Fluor (red) and DAPI (blue); (**b**) Values of cell cross-sectional areas (mean ± SEM) evaluated blind by two researchers using the drawing function of ImageJ software; immunolabelling and relative quantifications of α-smooth muscle actin (**c**,**d**), transgelin-2 (**e**,**f**), and β-catenin (**g**,**h**). Target proteins are stained in red, cell nuclei are in blue (DAPI). Quantifications of protein expression were performed blind by two researchers. All image magnifications are 20×; (**i**) immunoblotting (top) of myostatin detected in protein extracts of cultured hSCs and densitometric quantification (bottom). * *p* < 0.05, ** *p* < 0.01 and *** *p* < 0.001. In this experiment, hSCs were cultured for 24 h in a standard medium (M) or M supplemented with 100 nM Ang (M + Ang), or 1 µM irbesartan (M + Irb) or 1 µM Irb plus 100 nM Ang (M + Irb + Ang).

Since Ang is reported to stimulate hypertrophy and fibrosis in renal and cardiac cells [20], we explored whether similar effects were induced in activated hSCs. Cell conditioning with 100 nM Ang for 24 h resulted in increased cell cross-sectional areas, which were significantly larger compared to cells grown in a standard medium (M, Figure 4a,b, *** *p* < 0.001 vs. M). The effect was completely prevented by Irb, indicating that AT1 receptors were responsible for the hypertrophic response. Irb alone did not modify cell sizes, of which values were almost equal in cells grown in the M.

To assess whether sub-chroning conditioning with Ang also promoted a fate switch of hSCs toward myofibroblasts, we analyzed the expression of myofibroblast markers, namely α-smooth muscle actin (α-SMA) and transgelin-2. After 24 h conditioning with Ang, both markers were significantly increased compared to those in cells grown in the M (Figure 4c–f, α-SMA: *** *p* < 0.001 vs. M; transgelin-2: * *p* < 0.05 vs. M). Interestingly, expression levels remained high after cell conditioning with Ang plus Irb, suggesting that, differently from hypertrophy, myofibroblast trans-differentiation was independent of AT1 receptors. Similarly, β-catenin protein was increased in Ang- and Ang + Irb-conditioned cells (Figure 4g–h, ** *p* < 0.01 vs. M), further indicating an AT1-independent pathway leads to myofibroblast trans-differentiation.

Finally, we also analyzed the expression of myostatin, a potent pro-inflammatory and fibrogenic peptide acting as an inhibitor of myogenic differentiation. Surprisingly, blot analysis showed that myostatin levels were similar in the M, and Ang- and Irb-conditioned cells, while they significantly increased in cells conditioned with Ang plus Irb (Figure 4i; ** *p* < 0.01 vs. M). This further reinforced the hypothesis of an AT1 receptor-independent pathway supporting myofibroblast trans-differentiation.

We also assessed whether hypertrophy and trans-differentiation induced by Ang were associated to modifications of hSC viability. MTT oxidation values in all conditions expressed as mean ± standard deviation of the mean were similar: 0.090 ± 0.012 in cells grown in the M at t = 0 h, 0.167 ± 0.015 in cells grown in M for 24 h, 0.201 ± 0.005 in cells grown in Irb for 24 h, 0.143 ± 0.0085 in cells grown in Ang for 24 h, and 0.171 ± 0.01 in cells grown in Ang + Irb for 24 h. This finding excluded any negative effect of conditioning on cell viability.

### 2.4. Sub-Chronic Conditioning of Activated hSC with Ang Enhances TRPC Channel Function and Modifies Expression Patterns of AT Receptors

In order to provide a mechanistic hypothesis on the effects induced by Ang conditioning in activated hSCs, we assessed whether, similarly to renal and cardiac cells, a functional link between Ang and TRPC channels is effective in our model.

**Figure 5:**
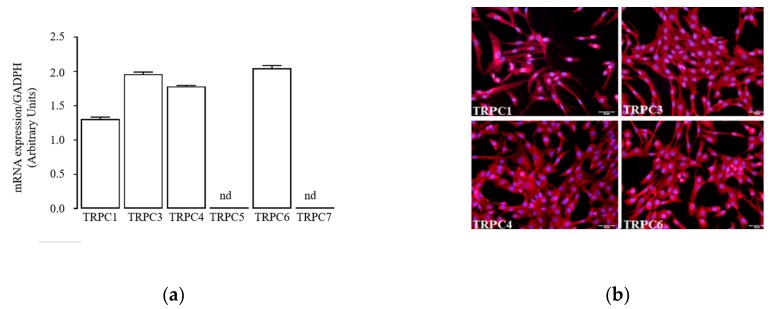

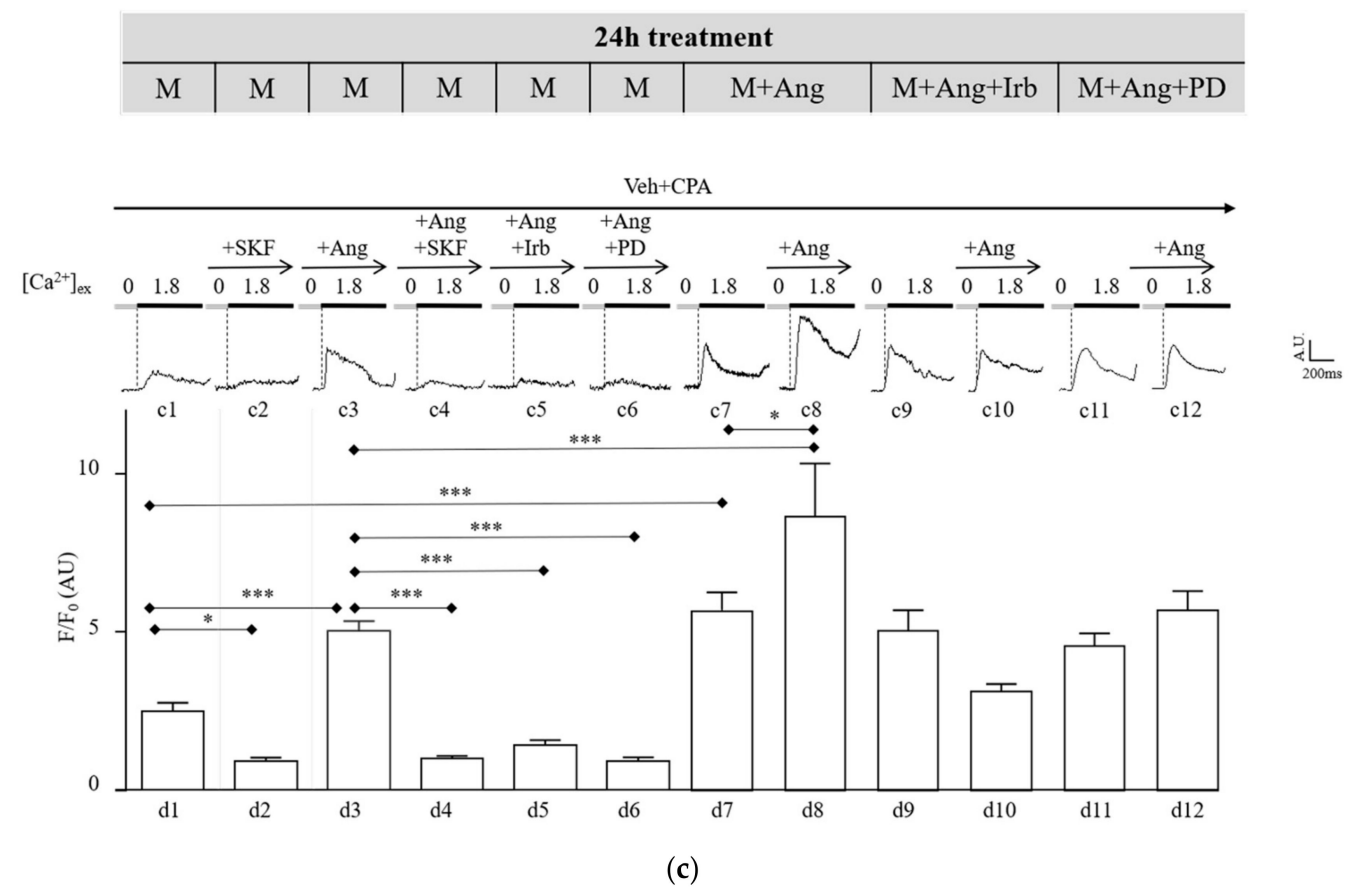
Functional coupling between Ang and TRPC channels on hSCs: (**a**) mRNA expression of TRPC1–7 channels in hSCs at passage 3; (**b**) immunolabelling of TRPC1, 3, 4, 6 channels in hSCs. Channel proteins are stained in red, and nuclei are in blue (DAPI). All image magnifications are 20X; (**c**) experimental scheme (top) used for 24 h conditioning of hSCs prior to fluorescence calcium imaging. Culture conditions were: M supplemented with 100 nM Ang (M + Ang), 100 nM Ang plus 1µM irbesartan (M + Ang + Irb), or 100 nM Ang plus 100 nM PD123319 (M + Ang + PD). The experimental protocol to perform fluorescence calcium imaging used a vehicle solution (Veh) supplemented with 5 µM cyclopentyladenosine (Veh + CPA) and 0 or 1.8 mM CaCl_2_ to stimulate a transient intracellular Ca^2+^ entry (trace c1). The histogram (bottom) shows mean peak values (±standard error) of Ca^2+^ transients measured in cells cultured in the M for 24 h (column d1), exposed to acute 5 µM SKF (d2, trace c2,), 100 nM Ang (d3, trace c3,), Ang plus SKF (d4, trace c4), Ang plus Irb (d5, trace c5), and Ang plus PD (d6, trace c6). Ca^2+^ transients measured in cells conditioned for 24 h in M + Ang (top scheme) are represented by trace c7 and column d7, and by trace c8 and column d8 after acute stimulation by 100 nM Ang. Similar measurements were performed in: cells conditioned for 24 h in M + Ang + Irb (top scheme) without/with acute stimulation by Ang (trace c9, column d9/trace c10, column d10); cells conditioned for 24 h in M + Ang + PD (top scheme) without/with acute stimulation by Ang (trace c11, column d11/trace c12, column d12). * *p* < 0.05, *** *p* < 0.001.

We first determined the expression panel of TRPC channels in activated hSCs by quantitative RT-PCR, immunofluorescence staining, and western blotting. Results revealed that TRPC1, 3, 4, 6 isoforms, but not TRPC5, 7, are detectable as mRNAs (Figure 5a) and as proteins in most cells (Figure 5b) and total protein extract (Figure S3).

Thereafter, we performed fluorescence calcium imaging in cultured hSCs and found that TRPC channels co-assembled into functional pores exhibiting properties consistent with those described for typical TRPC channels [25]. In detail, in cells grown for 24 h in the M (Figure 5c), depletion of intracellular Ca^2+^ stores by CPA (Veh + CPA), and switch from 0 to 1.8 mM external Ca^2+^ elicited a transient increase of intracellular fluorescence (Figure 5c, trace c1, and column d1), as expected for Ca^2+^ entry through the plasmalemma. A major component of it, accounting for nearly two-thirds of the total amount, was significantly inhibited by 5 µM SKF96365 (SKF, Figure 5c, trace c2, column d2, * *p* < 0.05 d2 vs. d1), a non-selective TRPC channel blocker. The residual SKF-insensitive component was not further investigated in the study.

The SKF-sensitive Ca^2+^-transient identified in our cell system was further characterized with respect to the effect of Ang. Acute exposure to 100 nM Ang for 10 min evidenced a positive modulation of TRPC channels (Figure 5c), trace c3 and column d3, *** *p* < 0.001 d3 vs. d1), indicating that these channels in hSCs share a typical modulatory signal identified in other cell types [25]. The enhancement was completely inhibited by SKF (Figure 5c), trace c4 and column d4, ***p < 0.001 d4 vs. d3), further confirming the involvement of TRPC channels in basal and Ang-stimulated Ca^2+^ entry into hSCs. The stimulatory effect of Ang was prevented by 1 µM Irb and 1 µM PD123319 (PD, Figure 5c), traces c5 and c6, columns d5 and d6, *** *p* < 0.001 vs. d3), selective antagonists of AT1 and AT2 receptors, respectively.

Next, to test whether subchronic conditioning with Ang leading to hypertrophy and myofibroblast trans-differentiation of hSC was associated to modifications of TRPC channel function, we repeated fluorescence calcium imaging using the same protocol described above in cells grown for 24 h in the M supplemented with 100 nM Ang, or Ang plus Irb, or Ang plus PD (Figure 5c), M + Ang, M + Irb, and M + PD). Results showed that in cells conditioned with Ang for 24 h, basal Ca^2+^ entry elicited by switching external Ca^2+^ from 0 to 1.8 mM was still evident and significantly higher compared to that measured in cells grown in the M for 24 h (Figure 5c), trace c7 and column d7, *** *p* < 0.001 vs column d1). Moreover, TRPC channels in Ang-conditioned cells still were stimulated by acute Ang, as demonstrated by the enhanced fluorescence (Figure 5c), trace c8 and column d8, * *p* < 0.05 vs column d7). Of note, the stimulatory effect of acute Ang was significantly larger compared to that induced in cells grown in the M for 24 h (Figure 5c), trace c8 and column d8, *** *p* < 0.001 vs. d3). Additionally, cells conditioned with Ang plus Irb or Ang plus PD for 24 h showed similar fluorescence levels that did not respond to acute Ang (Figure 5c), traces c9–c12 and columns d9–d12). Thus, TRPC channel function in hSCs is amplified by sub-chronic conditioning with Ang for 24 h both in the basal- and Ang-stimulated component.

**Figure 6:**
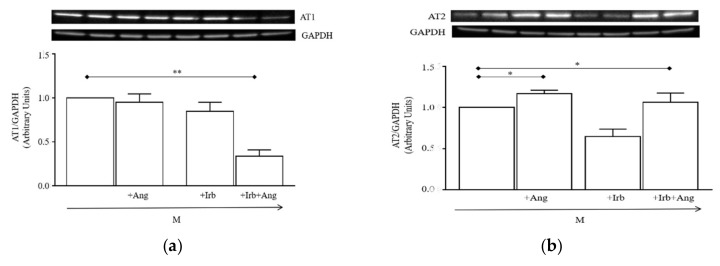
Effect of sub-chronic Ang conditioning of hSCs on expression of AT1 and AT2 receptors: (**a**) Representative immunoblotting (top) and densitometry analysis (bottom) of AT1 receptors expressed in hSCs cultured for 24 h in the M supplemented or not with 100 nM Ang, 1 µM Irb, or Ang plus Irb (Ang + Irb); (**b**) representative immunoblotting (top) and densitometry analysis (bottom) of AT2 receptors expressed in hSCs cultured as in (**a**). Densitometry analysis is reported as ratios between the levels of the target protein and GAPDH. Results are presented as mean ± SEM of the three gels. * *p* < 0.05 vs. M, ** *p* < 0.01 vs. M.

Finally, we assessed whether cell conditioning with Ang for 24 h modified the expression panel of AT receptors. Western blot analysis (Figure 6a,b) showed that AT1 receptors were not modified by 24 h conditioning with Ang or Irb alone, while they were significantly downregulated by Ang plus Irb compared to cells grown in M (** *p* < 0.01 vs. M). Differently, AT2 receptors were significantly upregulated by Ang conditioning (* *p* < 0.05 vs. M) and the enhancement was not prevented by Irb (* *p* < 0.05 vs. M).

## 3. Discussion

In healthy muscle, activated SCs balance myogenic differentiation and self-renewal to simultaneously sustain regeneration and replenish progenitor pools [1]. Latter events are major determinants to preserving long-term regenerative ability and effectively respond to growth stimuli. Any conditions able to exhaust SC pools, such as aging, cardiovascular diseases, and muscle dystrophies, may facilitate muscle wasting. Interestingly, all these conditions exhibit various degrees of RAS over-activation, elevated levels of systemic and local Ang, and muscle fibrosis [20].

Despite Ang is recognized as a potent fibrogenic factor in the muscle [2], little information is available on hSCs as cell mediators of fibrosis [33]. The main findings provided in this study demonstrate that Ang acts directly on activated hSCs, inducing hypertrophy and myofibroblast trans-differentiation in vitro (Figure 4), two crucial transitional states leading cells to enter the fibrotic program. This reprogramming switch integrates hSCs into the family of the direct cell sources of myofibroblasts in vitro and represents a novel finding for both the effects of Ang on myogenic cells and the cell sources of myofibroblasts [34]. Whether a similar reprogramming process occurs in vivo is currently unknown; indeed, most studies in muscle have documented the formation of myofibroblasts through differentiation of fibroblasts, reprogramming of bone marrow-derived cells, and mesenchymal transition of epithelial cells, disregarding the possibility of a direct myofibroblast trans-differentiation of SCs stimulated by Ang. Occurrence of such trans-differentiation in vivo would unveil a different cell source of fibrosis induced by locally elevated Ang, which could be relevant to identification of novel targetable mechanisms of muscle wasting.

Our data demonstrated that intracellular signaling elicited by Ang in activated hSCs, namely hyper-phosphorylation of p-mTOR, p-ERK1/2, p-P38, and p-AKT (Figure 3), resembles that involved in the hypertrophic response of cardiac cells by stimulation of Ang-AT1 [20]. This suggested that the hypertrophic effect of Ang in hSCs is likely attributable to the same AT1-signaling documented in cardiac cells. Conversely, our findings also showed that trans-differentiation of hSC into myofibroblasts is independent of AT1 receptors, as demonstrated by the inability of Irb to reduce fibrotic markers induced by cell conditioning with Ang (Figure 4c–i). This result led us to consider that other receptors expressed in our system, i.e., AT2 or MAS receptors, may play a role in the fibrogenic process. This possibility is not obvious and apparently opposes the anti-fibrotic role of AT2 and MAS receptors reported for skeletal muscle, heart, and kidney [20]. On the other hand, our experimental evidence is in line with data demonstrating a pro-fibrotic role of AT2 receptors when overexpressed in the heart [35,36,37]; notably, a similar expression pattern of Ang receptors is present in hSCs conditioned with Ang for 24 h, where AT2 receptors are increased, while AT1 are unchanged (Figure 6a,b).

A different explanation might attribute myofibroblast trans-differentiation induced by Ang to Ang-related peptides, i.e., Ang-(1–7) and Ang-(1–9), possibly formed in our culture system by the cleaving activity of ACE2. This enzyme is expressed at a protein level in hSCs (Figure 2); however, we did not explore the effective production of the above peptides at this time and it remains as an intriguing hypothesis to be tested in further work. Finally, another possibility is that Ang or Ang-(1–7) behave as biased agonists of AT1 receptors in hSCs [38], leading to the recruitment of β-arrestins and triggering of ERK1/2 phosphorylation; interestingly, p-ERK1/2 levels are enhanced in hSCs conditioned with Ang (Figure 3), which might thus make a contribution of this signal in our system.

Altogether, each of the above-mentioned hypotheses are likely to occur in our culture system; however, further investigations are necessary to clarify the major mechanism(s) occurring in our conditions.

Based on data supporting a role of Ang-TRPC channel axis in cardiac and renal fibrosis [23,39,40], in this study, we explored whether a similar role could be hypothesized in our setting. Indeed, we found that hSCs express TRPC1, 3, 4, 6 channel isoforms, which co-assembled into functional tetramers passing Ca^2+^ ions into hSCs. The Ca^2+^ flux was analyzed by fluorescence imaging and showed three peculiar properties, namely activation by depletion of internal Ca^2+^ stores, sensitivity to a selective blocker, and positive modulation by Ang (Figure 5c), which jointly are attributable to typical TRPC channels [25,41]. Interestingly, our findings demonstrated that TRPC channels in hSCs are stimulated by both AT1 and AT2 receptors. While the stimulatory effect by AT1 receptors is widely documented in the literature, that of AT2 receptors has only been reported in renal podocytes [42]. In these cells, the positive modulation exerted by AT1 and AT2 receptors on TRPC channels is part of a redundant pathway necessary to guarantee podocyte-specific functions in the kidney; it is possible that in hSCs such a double regulatory pathway is also required for basal function(s) in the muscle, as suggested by impaired myogenic differentiation in TRPC3 or STIM1/ORAI1 ablation models [30,43].

We also provided evidence that Ang sub-chronic conditioning of hSCs upregulates TRPC channel function, thereby amplifying basal and Ang-stimulated Ca^2+^ entry inside cells. This gain of function of TRPC channels is likely dependent on the increased expression of AT2 receptors (Figure 6) in hSCs conditioned with Ang, an effect that may be sufficient to enhance the positive coupling of Ang with TRPC channels. However, we cannot exclude other possible mechanisms, such as transcriptional upregulation of TRPC channels induced by Ang conditioning that might concur to enhance Ang-TRPC channel coupling. Regardless of the mechanism leading to enhance the Ang-TRPC channel axis in hSCs, we hypothesized that the amplified response of intracellular Ca^2+^ following these modifications may contribute to shaping the myogenic fate of hSCs, promoting their hypertrophy, and switching them toward myofibroblasts. This hypothesis is in line with experimental findings on fibrotic transformation of mouse C2C12 myoblasts induced by Ang, a transformation associated with enhanced intracellular Ca^2+^ levels, although this effect was not attributed to TRPC channels [44]. It is worth noting that many studies have documented that sustained intracellular Ca^2+^ levels in myogenic cells exert distinct long-term function through activation of downstream Ca^2+^-dependent signals, including nuclear factors of activated T-cells (NFAT), mitogen-activated protein (MAP) kinase, and ERK1/2 [30]. Importantly, signals arising from activation of the Ang-TRPC channel axis were proven to be key effectors of fibrosis in cardiac and renal cells [21,22,23,24,25,26,27], suggesting that this axis might represent a widespread promoter of fibrosis in tissues sensitive to abnormal levels of Ang.

In conclusion, our study provides evidence that Ang drives hSCs toward hypertrophy and myofibroblast differentiation, advancing current knowledge on RAS signaling on myogenic cells. Effects induced by Ang in hSCs are associated to basal- and Ang-stimulated TRPC channel gains of function, which we propose as possible molecular mediators of the effect of Ang. Altogether, this information represents a basis to investigate whether a similar reprogramming pathway of hSCs occurs in vivo in conditions of RAS over-activation. Overall, our findings support a potential benefit of RAS and/or TRPC channel blockers in muscle wasting and fibrosis.

## 4. Materials and Methods

### 4.1. hSC Preparation

The study was conducted in accordance with the Declaration of Helsinki 1975, revised in 2013. The protocol was approved by the Ethics Committees of Meyer Children’s Hospital, Florence, Italy (project protocol: 105/2016, date of approval: 28 June 2016), and of Azienda Ospedaliero-Universitaria Careggi, Florence, Italy (project protocol: 2015/0009082, date of approval: 25 March 2015). All subjects gave their informed consent for inclusion before they participated in the study.

Specimens from pectoralis muscle were obtained from waste skeletal muscle resection of patients undergoing corrective surgery at Meyer Children’s Hospital (Florence, Italy) using a protocol approved by the local ethics committee. Specimens were maintained in an appropriate cold transport solution (see Section 4.7) and cleaned of adipose tissue. Digestion was performed with Type 1 collagenase in high glucose DMEM (2.2 mg/mL, Sigma-Aldrich, Schnelldorf, Germany) at 37 °C for 30 min, in 5% CO2 and 90% humidified air. Digested fragments were then transferred to Promocell Growth Medium (Promocell Gmbh, Heidelberg, Germany) and gently pipetted until complete cell dispersion. Thereafter, cells were pre-plated in 6-well plates (5 × 10^2^ cells/well) for 1 h, at 37 °C, in atmosphere 5% CO2 and 90% humidified air, and non-adherent fibers were removed and plated on multi-wells coated with matrigel (Sigma-Aldrich, Schnelldorf, Germany). In this condition, fibers were maintained for 1–2 weeks, with the medium changed every 2 days, and evidenced SC proliferation within 2 weeks. Proliferating SCs were then cultured at 60%–70% confluence, detached by standard trypsinization and diluted 1:3. All subsequent experiments were performed at passages 1–3.

### 4.2. Immunofluorescence

hSCs were seeded in a 12-well plate (5 × 10^2^ cells/well) and maintained in a standard growing medium (CTR), supplemented or not with Ang (0.1, 1, or 100 nM), Irb (1 µM), or Ang (100 nM) and Irb (1 µM). The durations for drug exposure were 15 min and 24 h in acute and sub-chronic treatments, respectively. Before staining, hSCs were washed with PBS, fixed with 4% paraformaldehyde in PBS for 15 min at room temperature, washed again, and permeabilized with 0.3% TritonX100 in PBS for 10 min. Thereafter, cells were treated for 10 min at room temperature with PBS containing 1% BSA to avoid non-specific binding of antibodies. Primary antibodies (Table 1), diluted in PBS containing 0.1% Tween and 1% BSA, were incubated overnight at 4 °C; after washing with PBS, treatment with the secondary antibody (Table 1, diluted 1:150) was performed for 2 h at room temperature. Finally, hSCs were treated with 0.1% Tween and 4′,6-diamidin-2 phenylindole (DAPI; 0.005 µg/mL PBS and images viewed by a low-power objective (20X magnificationand a rhodamine filter. Images were digitized using a video image obtained by a CCD camera (Diagnostic Instruments Inc., Sterling Heights, MI, USA), controlled by a specific software (InCyt Im1™; Intracellular Imaging Inc., Cincinnati, OH, USA), and analyzed using ImageJ 1.33 analysis software. For each target, 20 fields were taken randomly. All reagents for immunofluorescence, unless otherwise specified, were purchases from Sigma-Aldrich (Schnelldorf, Germany).

### 4.3. Western Blot Analysis

Protein lysates were recovered from plated hSCs using lysis buffer and analyzed by the BCA method (Pierce). Twenty micrograms of proteins were separated on 4%–20% SDS-PAGE (Thermo Fisher scientific, Life Technologies, Carlsbad, CA, USA) and transferred into PVDF membranes (60 min at 398 mA) using standard procedures. Blots were incubated overnight at 4 °C with a specific antibody (Table 1). Primary antibodies were diluted in PBS containing 1% BSA or 5% non-fat dry milk and 0.05% Tween. The antigen–antibody complexes were visualized using appropriate secondary antibodies (diluted 1:10000 in PBS containing 1% albumin or 5% non-fat dry milk and 0.05% Tween) and left for 1 h at room temperature. Blots were then extensively washed with PBS containing 0.1% Tween and developed using an enhanced chemiluminescence detection system (Pierce, Rodano, Italy). Exposure and developing time were standardized for all blots. Densitometric analysis of scanned images was performed on a Macintosh iMac computer using the public domain NIH Image program. Results represent the mean ± SEM of different gels and are expressed as arbitrary units (AUs), consisting of the ratio between the levels of target protein and GAPDH. All reagents, unless otherwise specified, were purchases from Sigma-Aldrich (Schnelldorf, Germany).

### 4.4. Quantitative PCR Analysis

Total RNA was obtained from cultured hSCs using the Macherey Nagel Nucleo spin RNA kit (MACHEREY-NAGEL GmbH & Co. KG, Düren, Germany). One microgram of total RNA was reversed transcribed using iScript™ Select cDNA Synthesis Kit (Bio-Rad Laboratories, Bio-Rad Laboratories, Segrate (MI), Italy), according to the manufacturer’s instructions. Gene primers were obtained from Bio-Rad Laboratories Bio-Rad Laboratories, Segrate (MI), Italy: TRPC1 (unique assay ID: qHsaCED0045239), TRPC3 (unique assay ID: qHsaCID0018563), TRPC4 (unique assay ID: qHsaCED0036540), TRPC5 (unique assay ID: qHsaCID0017182), TRPC6 (unique assay ID: qHsaCED0034676), TRPC7 (unique assay ID: qHsaCID0009550), and GADPH (unique assay ID: qAthCED0035454). Real-time PCR reaction was carried out in 10 μL volumes in 96-well plates (Applied Biosystems, London, UK) using 1 μL of cDNA mixed with 3 μL nuclease-free water, 1 μL of sense and antisense primers (10 μM), and 5 μL of Ssoadvanced Universal SYBR Green Supermix (Bio-Rad Laboratories, Segrate (MI), Italy). Reaction was performed using the following thermocycler (7900HT Fast Real-Time PCR System (Applied Biosystems, London, UK) program for all genes: 95 °C for 1 min followed by 40 cycles at 95 °C for 15 s, at 60 °C for 60 s, and at 72 °C for 45 s. Each experiment was repeated in triplicate, and quantitative analysis was performed with the ΔCT method. GAPDH was used for normalization. As a positive control, a homogenized sample from human kidney was used (Figure S2).

### 4.5. Assessment of Cell Viability/Growth

hSCs were plated at a density of 500 cells/plate in 96-well plates and cultured in standard conditions for 24 h and then in a starving medium (i.e., DMEM with 0.01% BSA) for additional 24 h. Thereafter, cells were exposed to a complete growing medium (CTR) with or without Ang (100 nM) and Irb (1 µM). Cell growth was evaluated 24 h later by measuring MTT oxidation (Sigma-Aldrich, Schnelldorf, Germany).

### 4.6. TRPC-Mediated Ca^2+^ Imaging

Adherent hSCs were loaded with 5–10 µM Fluo-4 AM (Molecular Probes, Eugene, OR, USA) and placed in a heated (36 ± 1 °C) stage of an inverted microscope. Experimental protocols were similar to those described earlier [25]. Briefly, cells were perfused for 10–15 min with a Ca^2+^-free normal Tyrode’s solution (see Section 4.7) containing cyclopiazonic acid (CPA, 5 µmol/L) to deplete intracellular Ca^2+^ stores. Cells were then switched to the same solution supplemented with 1.8 mM CaCl_2_ to stimulate TRPC-mediated Ca^2+^ entry. An increase in Fluo-4 fluorescence intensity following external Ca^2+^ rise was measured and compared to baseline fluorescence. Ang (100 nM), Irb (1 µM), PD123319 (1 µM), or the TRPC channel inhibitor SKF96365 (5 µM) were diluted in Tyrode’s solution containing CPA with or without 1.8 mmol/L CaCl_2_. All reagents, unless otherwise specified, were purchases from Sigma-Aldrich (Schnelldorf, Germany).

### 4.7. Solutions

Transport solution (mmol/L): KH_2_PO_4_ 50, MgSO_4_ 8, HEPES 10, adenosine 5, glucose 140, mannitol 100, and taurine 10 (pH 7.4 with KOH).

Tyrode’s solution (mmol/L): NaCl 132, KCl 4, MgCl_2_ 1.2, HEPES 10, and glucose 11 (pH 7.35 with NaOH). All reagents were purchases from Sigma-Aldrich (Schnelldorf, Germany).

### 4.8. Statistical Analysis

All data are expressed as mean ± SEM. Statistical analysis was performed by one-way ANOVA followed by post-hoc analysis or unpaired Student’s *t*-test when appropriate. A *p*-value of <0.05 was taken to indicate statistical significance.

## Figures and Tables

**Table 1:** List of primary and secondary antibodies used for immunofluorescence (IF) and western blot (WB) analysis.

Antibodies	Dilution	Supplier
Anti-ACE1	1:50 (IF)	Santa Cruz Biotechnology, Heidelberg, Germany
Anti-ACE2	1:50 (IF)	Santa Cruz Biotechnology, Heidelberg, Germany
Anti-ATR1	1:100 (IF) 1:500 (WB)	Alomon Labs, Jerusalem, Israel
Anti-ATR2	1:200 (WB)	Alomon Labs, Jerusalem, Israel
Anti-GAPDH	1:7000 (WB)	Merk-Millipore, Darmstadt, Germany
Anti-Myf5	1:100 (IF)	Santa Cruz Biotechnology, Heidelberg, Germany
Anti-MyoD	1: 100 (IF)	Santa Cruz Biotechnology, Heidelberg, Germany
Anti-myogenin	1:100 (IF)	Developmental Studies Hybridoma Bank, Iowa City, Iowa, United States of America
Anti-myostatin	1:300 (WB)	Biorbyt Explore, Cambridge, United Kingdom
Anti-myosin heavy chain	1:100 (IF)	BD Pharmingen, San Jose, California, United States of America
Anti-Pax7	1:100 (IF)	Developmental Studies Hybridoma Bank, Iowa City, Iowa, United States of America
Anti-p-AKT	1:1000 (WB)	Cell Signaling Technology, Leiden, The Netherlands
Anti-p-ERK1/2 (Thr202/Tyr204)	1:1000 (WB)	Cell Signaling Technology, Leiden, The Netherlands
Anti-p-mTOR	1:1000 (WB)	Cell Signaling Technology, Leiden, The Netherlands
Anti-p-P38	1: 400 (WB)	Cell Signaling Technology, Leiden, The Netherlands
Anti-TRPC1	1:100 (IF) 1:200 (WB)	Alomon Labs, Jerusalem, Israel
Anti-TRPC3	1:100 (IF) 1:200 (WB)	Alomon Labs, Jerusalem, Israel
Anti-TRPC4	1:100 (IF) 1:200 (WB)	Alomon Labs, Jerusalem, Israel
Anti-TRPC5	1:100 (IF) 1:200 (WB)	Alomon Labs, Jerusalem, Israel
Anti-TRPC7	1:100 (IF) 1:200(WB)	Alomon Labs, Jerusalem, Israel
Anti-Mas receptor	1:100 (IF)	Santa Cruz Biotech, Heidelberg, Germany
Anti-smooth muscle actin	1:100 (IF)	Dako, Santa Clara, California, United States of America
Anti-catenin	1:1000 (IF)	BD Pharmingen, San Jose, California, United States of America
Anti-transgelin 2	1:100 (IF)	Everest Biotech, Upper Heyford, United Kingdom
Anti-vimentin	1:100 (IF)	Santa Cruz Biotech, Heidelberg, Germany
Anti-rabbit IgG-Alexa Fluor 594	1:500 (IF)	Thermo Fisher scientific, Bengaluru, India
Anti-chicken IgG-Alexa Fluor 647	1:500 (IF)	Thermo Fisher scientific, Bengaluru, India

## Supplementary Materials

Supplementary materials can be found at https://www.mdpi.com/1422-0067/20/19/4912/s1.

## Abbreviations

SC	satellite cell
hSC	hSC
Ang	angiotensin-II
RAS	renin angiotensin system
TRPC	transient receptor potential canonical
AT1R	Ang type-1 receptor
AT2R	Ang type-2 receptor
ACE	angiotensin-converting enzyme
ACEi	angiotensin-converting enzyme inhibitors,
ARB	AT1 receptor antagonist
Pax7	paired box protein 7
Myf5	myogenic factor 5
MyoD	myogenic factor 3
Myogenin	Myogenin
MHC	myosin heavy chain
ACE1	type-1 angiotensin-converting enzyme
ACE2	type-2 angiotensin-converting enzyme
Irb	Irbesartan
α-SMA	α-smooth muscle actin
