# Supplementary material for: Angiotensin-II Drives Human Satellite Cells Toward Hypertrophy and Myofibroblast Trans-Differentiation by Two Independent Pathways
Source: Int J Mol Sci. 2019 Oct 3;20(19):4912. doi: 10.3390/ijms20194912 (PMC6801484; doi:10.3390/ijms20194912)
Supplement: Supplementary file 1 [file ijms-20-04912-s001.pdf]

International Journal of *Molecular Sciences*

MDPI

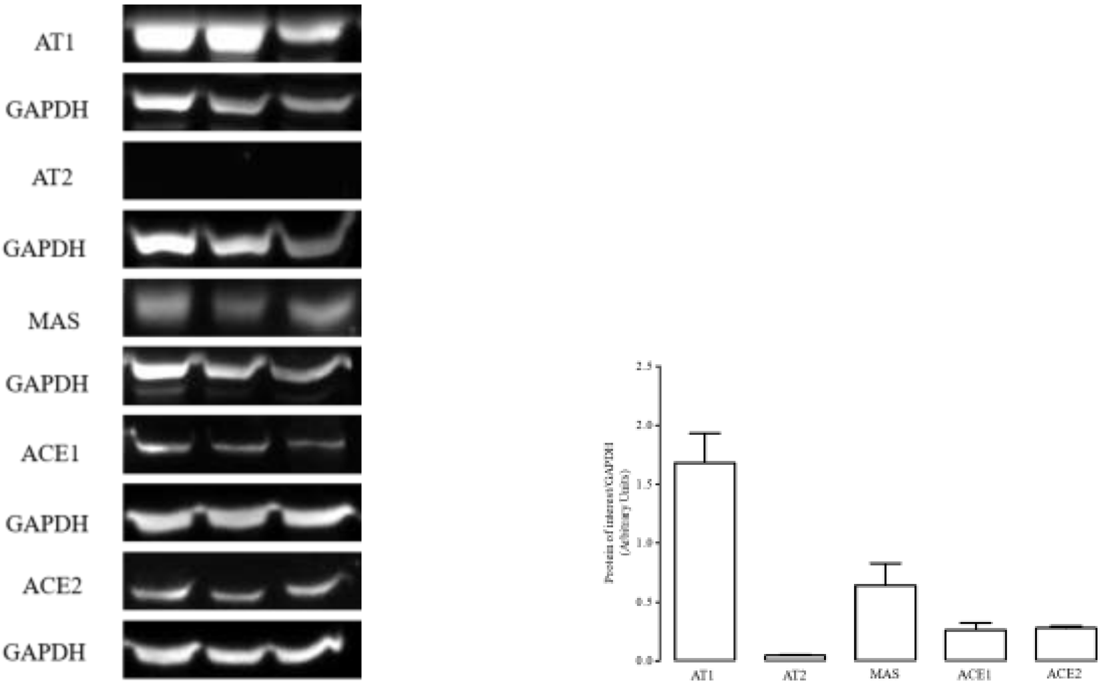

(a) **(b)**

Figure S1. Expression of RAS proteins in whole human skeletal muscle. Intact specimens of human skeletal muscle were homogenized and processed for Western blot analysis of RAS proteins. Representative immunoblotting (**a**) and densitometric analysis (**b**) of type-1 (AT1), type-2 (AT2) and Mas (Mas) receptors, angiotensin converting enzyme 1 (ACE1) and 2 (ACE2). Histogram shows the ratios (mean±standard error of the mean (SEM), n=3) between the expression levels of target proteins and GAPDH.

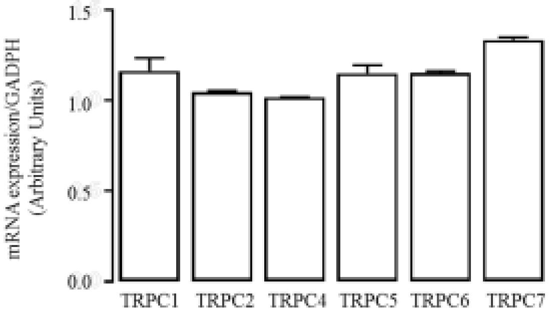

Figure S2. mRNA expression of TRPC channel isoforms in human kidney. Total RNA was obtained from human kidney sample and processed as described in methods for quantitative expression (relative to GADPH) of TRPC(1-7) channel isoforms.

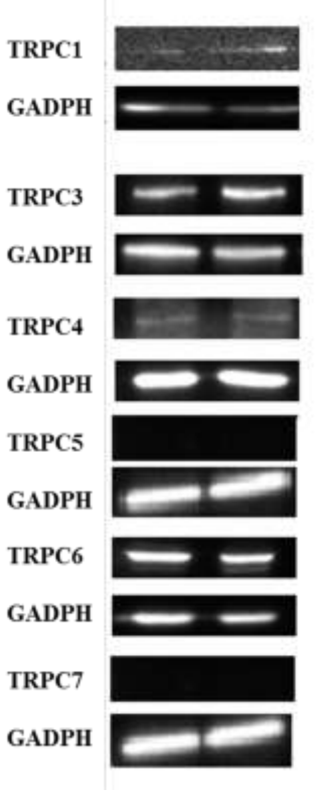

Figure S3. Expression of TRPC proteins in total protein extract of activated hSCs. Total protein of hSCs was processed for Western blot analysis of TRPC proteins. Representative immunoblotting of TRPC channel isoforms (1-7) and GADPH.

© 2019 by the authors. Submitted for possible open access publication under the terms and conditions of the Creative Commons Attribution (CC BY) license (http://creativecommons.org/licenses/by/4.0/).
